# The lectin-like oxidized LDL receptor-1: a new potential molecular target in colorectal cancer

**DOI:** 10.18632/oncotarget.7430

**Published:** 2016-02-17

**Authors:** Michela Murdocca, Ruggiero Mango, Sabina Pucci, Silvia Biocca, Barbara Testa, Rosamaria Capuano, Roberto Paolesse, Massimo Sanchez, Augusto Orlandi, Corrado di Natale, Giuseppe Novelli, Federica Sangiuolo

**Affiliations:** ^1^ Department of Biomedicine and Prevention, Tor Vergata University, Rome, Italy; ^2^ Department of Emergency and Critical Care, Section of Cardiology, Policlinic of Tor Vergata, Rome, Italy; ^3^ Department of Systems Medicine, Tor Vergata University, Rome, Italy; ^4^ Department of Electronic Engineering, Tor Vergata University, Rome, Italy; ^5^ Department of Chemical Science and Technology, Tor Vergata University, Rome, Italy; ^6^ Department of Cell Biology and Neurosciences, Istituto Superiore di Sanità, Rome, Italy

**Keywords:** colon cancer, LOX-1, VOCs analysis, shRNAs

## Abstract

The identification of new biomarkers and targets for tailored therapy in human colorectal cancer (CRC) onset and progression is an interesting challenge.

CRC tissue produces an excess of ox-LDL, suggesting a close correlation between lipid dysfunction and malignant transformation. Lectin-like oxidized LDL receptor-1 (LOX-1) is involved in several mechanisms closely linked to tumorigenesis.

Here we report a tumor specific LOX-1 overexpression in human colon cancers: LOX-1 results strongly increased in the 72% of carcinomas (*P*<0.001), and strongly overexpressed in 90% of highly aggressive and metastatic tumours (*P*<0.001), as compared to normal mucosa. Moreover LOX-1 results modulated since the early stage of the disease (adenomas vs normal mucosa; *P*<0.001) suggesting an involvement in tumor insurgence and progression.

The *in vitro* knockdown of LOX-1 in DLD-1 and HCT-8 colon cancer cells by siRNA and anti-LOX-1 antibody triggers to an impaired proliferation rate and affects the maintenance of cell growth and tumorigenicity. The wound-healing assay reveals an evident impairment in closing the scratch. Lastly knockdown of LOX-1 delineates a specific pattern of volatile compounds characterized by the presence of a butyrate derivative, suggesting a potential role of LOX-1 in tumor-specific epigenetic regulation in neoplastic cells.

The role of LOX-1 as a novel biomarker and molecular target represents a concrete opportunity to improve current therapeutic strategies for CRC. In addition, the innovative application of a technology focused to the identification of LOX-1 driven volatiles specific to colorectal cancer provides a promising diagnostic tool for CRC screening and for monitoring the response to therapy.

## INTRODUCTION

Colorectal cancer (CRC) is currently one of the principal causes of deaths in the Western countries [1]. Increased CRC risk is associated with obesity, diabetes, high cholesterol and atherosclerosis, which are components of a disease state known as “metabolic syndrome” [2, 3]. Importantly, all of these pathological conditions are associated with an increased formation of oxygen-reactive species (ROS). Continuous exposure to free radicals promotes oxidative damage to lipids, proteins and DNA, contributing to cell transformation [4]. However, few epidemiologic studies have investigated the relationships between lipid peroxidation and colorectal cancer [5–7]. Between these, one revealed that individuals with high levels of circulating oxidized low-density lipoprotein (ox-LDL), in addition to atherosclerotic plaques, are more prone to develop colorectal cancer. Moreover, CRC tissue produces and accumulates an excess of ox-LDL, suggesting a close correlation between lipid dysfunction and malignant transformation. This hypothesis is further supported by the observation that inhibition of cholesterol production by statins protects against cancerogenesis [8] and that among patients with newly diagnosed coronary artery disease the prevalence of CRC was greater [9].

Hirsh and colleagues [2] showed that a number of lipid metabolic genes are consistently overexpressed in diverse cancer cell lineages and, importantly, the expression of these genes is critical to cellular transformation, as well as in maintaining the transformed state. Strikingly, among these genes, *orl1*, encoding for the lectin-like oxidized LDL receptor-1 (LOX-1), emerges as the most relevant. *Orl1* gene is located on human chromosome 12p13.2-13.1 [10] and various polymorphisms (SNPs) have been characterized as playing a role in cardiovascular diseases susceptibility [11, 12]. LOX-1 is expressed in endothelial cells (aortic, carotid, thoracic, coronary arteries, veins), in macrophages, smooth muscle cells (SMC), fibroblasts and platelets [13]. The basal expression of LOX-1 is low, but it is up-regulated in pathological conditions affecting the cardiovascular system (i.e. hypertension, diabetes) and it plays an important role in the development of atherosclerosis [14, 15].

LOX-1 is the major receptor for ox-LDL in endothelial cells. It is a type II transmembrane glycoprotein belonging to the C-type lectin family and contains four domains: a short N-terminal cytoplasmic domain, a transmembrane domain, a neck domain and a lectin-like extracellular C-terminal domain (CTLD) [16–18]. The CTLD domain, which interacts with ox-LDL, forms a disulfide-linked heart-shaped homodimer, which assembles in larger functional oligomers through non covalent interactions [12, 19–20]. LOX-1 receptors are distributed within caveolae/lipid rafts in the plasma membranes and chronic exposure of cells to statins leads to a spatial disorganization of LOX-1 and a marked loss of LOX-1 function [21]. Notably, we have recently shown that statins, besides their indirect effect on LOX-1 activity derived from lowering intracellular cholesterol, inhibit LOX-1 by a direct interaction with the CTLD recognition domain, indicating a new previously unrecognized pleiotropic effect of this class of drugs [22].

Ox-LDL binding to LOX-1 increases reactive oxygen species (ROS) formation, strongly contributing to oxidative DNA damage that can be abrogated by LOX-1 inhibition [23]. ROS cause *in vivo* oxidation of lipids, proteins and DNA; recent studies have highlighted a positive correlation between increased levels of free radicals and lipid peroxides and carcinogenesis [5, 6]. Furthermore, ox-LDL binding to LOX-1 reduces the release of nitric oxide (NO) with the activation of NF-kB in endothelial cells [24, 25].

In particular, the depletion of LOX-1 receptors protects against tumorigenicity, motility and growth of these cells. These beneficial effects exerted by LOX-1 depletion are common among several lineages, such as hepatocellular carcinoma, breast and cervical cancers [2].

The meta-analysis of gene expression profiles of about 950 cancer cell lines stored in the Gene Expression Atlas at the EMBL-EBI database (http://www.ebi.ac.uk/gxa/gene/ENSG00000173391#) reveals that *olr1* is upregulated in 57% of bladder and cervix cancer cells, 11% of mammary gland cancer cells, 10% of lung cancer cells and importantly in 20% of CRC cells. Furthermore, a strong correlation between serum level of ox-LDL and risk of colorectal cancer was described in a large-scale Japanese cohort [26].

In this study we analyzed LOX-1 expression in different steps of human colon tumorigenesis and observed some features of neoplastic phenotype in colon cancer cell lines upon altering LOX-1 expression level. We used a shRNA-expressing lentiviral vector targeting the mRNA encoded by the *orl*1 gene in two colorectal cancer cell lines to down-regulate LOX-1 expression and analyzed changes in cell growth, motility and release of volatile organic compounds.

## RESULTS

### LOX-1 is overexpressed in human colon cancer

**Figure 1:**
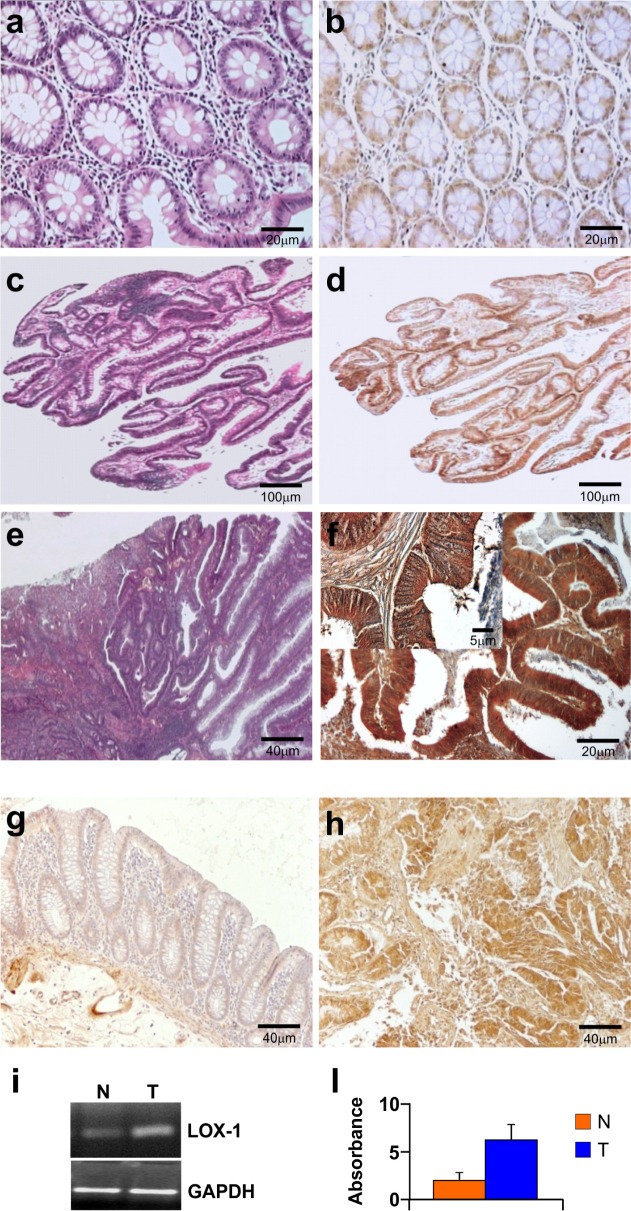
Immunohistochemistry and RNA expression of LOX-1 in colorectal cancer patients Haematoxyline eosin counterstain of **a.** normal mucosa, **c.** low grade adenoma, **e.** adenocarcinoma; **b, d, f, g, h**: immunohistochemistry analysis of LOX-1. **b.** A faint positive signal of LOX-1 was observed in normal mucosa (magnification 20X) **d**. A low-power field (magnification 4x) of adenoma. A diffuse positive signal was observed in low grade adenoma. **f** A low-power magnification (20X) of G2;pT3;N1 CRC patient. Higher magnification (40X) in the inset. **h.** LOX-1 overexpression in a G2;pT2;N0 CRC patient, as compared to (**g**) distant healthy mucosa of the same patient (magnification 10X); **i.** LOX-1 expression by RT-PCR from normal (lane **N**) and tumoral tissues (lane **T**) of the same patient. **l.** Values in the graph are relative to GAPDH normalization and are representative of three independent experiments.

Immunohistochemistry was performed to verify whether LOX-1 expression was related to neoplastic transformation from healthy tissue to adenoma and carcinoma. Colon carcinomas were characterized by grading and staging according to WHO and TNM classifications. Table 1 (a and b) summarize the main clinical and histological features correlated to LOX-1 expression evaluated by immunohistochemistry, as described in Materials and Methods section. As shown in Figure 1, LOX-1 expression was related to the tumour stage and grade: its expression in the colonic mucosa of healthy subjects (8 cases), collected from autoptic examination, and in the normal mucosa aside from the neoplasia (28 cases) was faint and related to cytoplasmic compartment (Figure 1b). In 6 out of 8 low-grade colonic adenomas LOX-1 expression was positive and resulted increased as compared to normal mucosa (*P*<0.001), as summarized in Table 1b, suggesting its potential involvement in the early stage of the disease (Figure 1d). Low grade carcinomas displayed a substantial increase of LOX-1 expression in the cytoplasm, whereas no positive staining was detected in the nucleus counterstained with haematoxylin. Moreover LOX-1 expression resulted strongly increased in the 72% of carcinomas (G1-G2;any T;N0), and strongly overexpressed in 90% of highly aggressive and metastatic tumours, as compared to normal mucosa (G2-G3;any T;N1,N2,M0,M1 vs NM: *P*< 0.001) (Figure 1f and Table 1b). It is worth of note that LOX-1 was increased in the transition from normal to neoplastic phenotype in the colon adenomas, suggesting a potential involvement in the early stage of the disease (ALGD vs NM *P*<0.001; tab 1b). Figure 1 a,c,e showed representative images of normal mucosa, low grade adenoma and adenocarcinoma respectively, counterstained by haematoxyline eosin. To evaluate LOX-1 gene expression in healthy and tumoral tissues, RNA was extracted from fresh tumoral (G2, pT2, N0) and healthy tissues of the same patient, as previously described. LOX-1 level was evaluated by immunohistochemistry in the same tissue used for RNA expression in order to compare the level of transcript to protein expression. As shown in Figure 1, LOX-1 was upregulated in pathological tissues (Figure 1h), as compared to normal mucosa aside from the neoplasia of the same patient (Figure 1g), confirming a tumor specific modulation of LOX-1 expression in colon cancer. As shown in Figure 1i and 1l, LOX-1 mRNA evaluated by RT-PCR, in tumoral tissue was increased 3 times, as compared to the healthy counterpart, corroborating immunohistochemical observations.

**Table 1a:** Clinical and pathologic factors of patients (n=28) with colorectal carcinoma

Factors	Patients (n=28)
**Location of tumour**	
Colon right	5
Colon left	21
Rectum	2
**Histology**	
Well-Moderately differentiated	18
Poorly differentiated	10
**TNM stage**	
T1	2
T2	10
T3	13
T4	3
**Lymphonodemetastasis**	
N0	18
N1-2	10
**Duke's stage**	
A	0
B	18
C	7
D	3

**Table 1b:** LOX- 1 protein expression, detected by immunohistochemistry, in human tumors and controls

	A	B	C	D
	**CRC**	**CRC**	**ALGD**	**NM**
	G2-G3; any T;	G1-G2; any T; N0		
	N1,N2, M0, M1			
	% (n=10)	% (n=18)	% (n=8)	% (n=36)
**LOX-1**				
Neg Neg/Weak	0	0	25(2)	94.4(34)
Pos Moderate	10(1)	23(5)	75(6)	5.5(2)
Strong	90(9)	72(13)	0	0

Number of cases was shown as a percentage. In parentheses, the absolute numbers were reported.

CRC: colorectal adenocarcinomas; ALGD: adenomas with low-grade dysplasia. Distant normal mucosa (NM) of the same patients was used as control.

Significance all data *P*_tot_<0.001; A vs D: *P*<0.001; B vs D: *P*<0.001; C vs D: *P*<0.001

**Figure 2:**
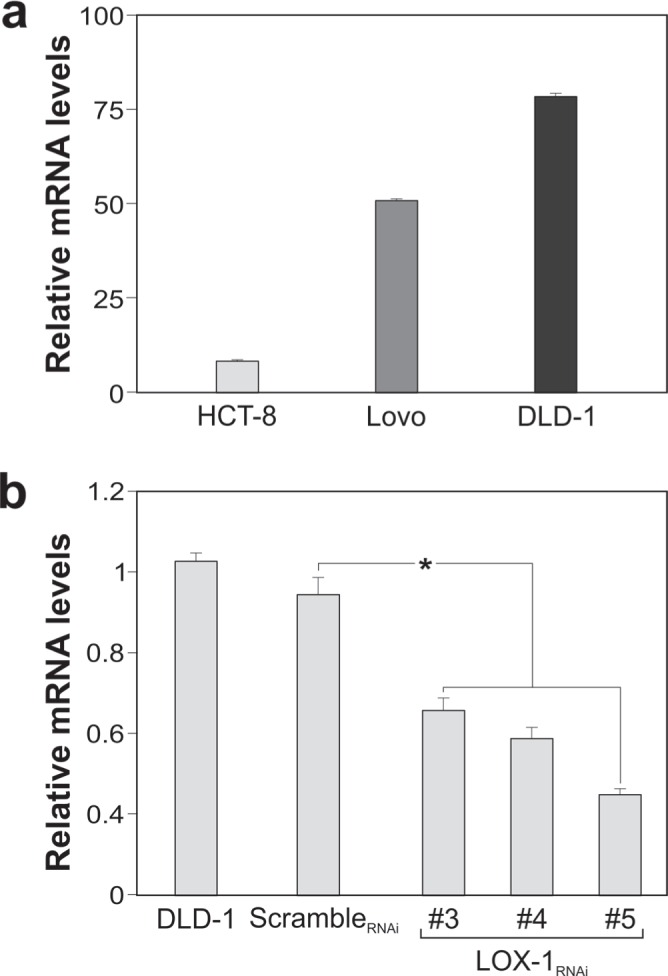
LOX-1 expression in colon carcinoma cell lines **a.** LOX-1 mRNA expression in SW480, HCT-8, LoVo and DLD-1cancer cells quantified by RT–qPCR. Values obtained are compared to SW480, a colorectal adenocarcinoma randomly selected among cells expressing LOX-1, and considered as unit. GAPDH are used as reference gene. Data are representative of three independent experiments and reported as mean±SEM. **b.** RT-qPCR for evaluating LOX-1 expression following lentiviral transduction and puromycin selection in DLD-1 cells infected by scramble_RNAi_ and by distinct LOX-1_RNAi_ (#3,#4,#5) designed against different regions of LOX-1 mRNA. Data are representative of three independent experiments and reported as mean±SEM (*P<0.05).

In order to better clarify the role of LOX-1 in colon tumorigenesis and its relation with the aggressiveness of the tumor, we performed *in vitro* studies on colon carcinoma cell lines deriving from primary tumors with different grades and stages (see Materials and Methods). To do this we assessed the relative expression levels of *orl1* mRNA in SW480, HCT8, LoVo, and DLD-1 cell lines, as shown in Figure 2a. LOX-1 expression levels were compared to those obtained in SW480 adenocarcinoma cell line, selected among those expressing LOX-1. Two cell lines expressing high levels of LOX-1 were chosen: metastatic DLD-1 cell line and non metastatic HCT-8 cell line.

### LOX-1 knockdown by RNAi

**Figure 3:**
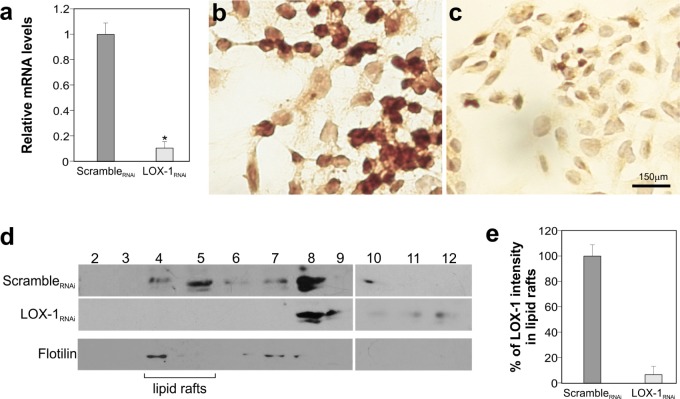
LOX-1 inhibition by shRNA in DLD-1 cells **a.** Real time-qPCR analyses of LOX-1 mRNA levels in DLD-1 cells 6 days post virus administration. Data are representative of three independent experiments and reported as mean±SEM (*P <0.05). **b**. Immunocitochemistry of LOX-1 expression of DLD-1 transduced by scramble_RNAi_ and **c.** by LOX-1_RNAi._; magnification 40X. **d**. Detergent-free purification of caveolin-rich domains by sucrose gradient. An aliquot of each fraction, collected from the top to the bottom of the gradient, was subjected to immunoblot analysis carried out with anti-LOX-1 and anti-flotilin antibodies. The numbers were referred to different fractions; in particular fractions 4 to 5 were designated as lipid rafts indicated by the marker protein flotilin. **e.** Histogram shows the densitometric measurements performed to compare the intensity of LOX-1in lipid raft fractions (fractions 4 and 5) derived from scramble_RNAi_ and LOX-1_RNAi_ DLD-1 cells. The data represent the average ± S.D. of three separate experiments.

To knockdown LOX-1 mRNA, we developed a strategy to obtain bi-directional lentiviral vectors expressing short hairpin RNAs (shRNAs) against mRNA. Four different shRNAs, including a scramble shRNA not targeting any human gene, were designed and tested in DLD-1 cell line. The analysis carried out by Real-Time qPCR, following transduction of DLD-1 cells, is reported in Figure 2b. Among shRNAs analyzed, the target sequence #5 (LOX-1_RNAi_; specifically designed against LOX-1 exon 4) produced the best results and, thus, it was chosen for LOX-1 inhibition. The degree of LOX-1 knockdown, following lentiviral delivery of LOX-1_RNAi,_ was compared to a scramble_RNAi_ and evaluated after infection. As shown in Figure 3a, six days after virus administration and antibiotic selection, the level of LOX-1 mRNA was reduced by approximately 85%. Immunocitochemistry on DLD-1 cells was performed to evaluate the inhibition efficiency. A strong expression of LOX-1 was observed by immunocitochemistry in scramble_RNAi_ DLD-1 (Figure 3b). An effective inhibition of LOX-1 expression was shown in LOX-1_RNAi_ DLD-1 cells (Figure 3c), confirming the silencing efficiency.

LOX-1 knockdown was also analyzed at protein level, by isolating caveolin-rich domains of DLD-1 cells by a detergent-free procedure and sucrose gradient flotation centrifugation (Figure 3d) [21]. An aliquot of each fraction, collected from the top to the bottom of the gradient, was subjected to immunoblot analysis (Figure 3d; fractions from 2 to 12). This procedure allowed us to compare LOX-1 expression levels in scramble_RNAi_ respect to LOX-1_RNAi_ DLD-1 cells and to study the intracellular distribution of LOX-1 receptors in cancer cells. As it can be seen, LOX-1 is partly found in fractions 4-5 in scramble_RNAi_ DLD-1 cells. The rest of LOX-1 receptors distributes in heavier vesicular fractions. Fractions 4 and 5 are composed of lipid rafts, as confirmed by the presence of flotilin-1 in the blot visualized by anti-flotilin-1 antibodies (Figure 3d). Of note, there is a marked reduction of LOX-1 band in LOX-1_RNAi_ DLD-1, especially in lipid rafts fractions, where LOX-1 is barely visible. From a densitometric analysis of band intensity from different experiments, gradients derived from LOX-1_RNAi_ DLD-1 cells present a reduction of 95±6 % of LOX-1 band in fractions 4-5 (Figure 3e).

### LOX-1 mRNA knockdown affects cell growth and motility of colon cancer cells

**Figure 4:**
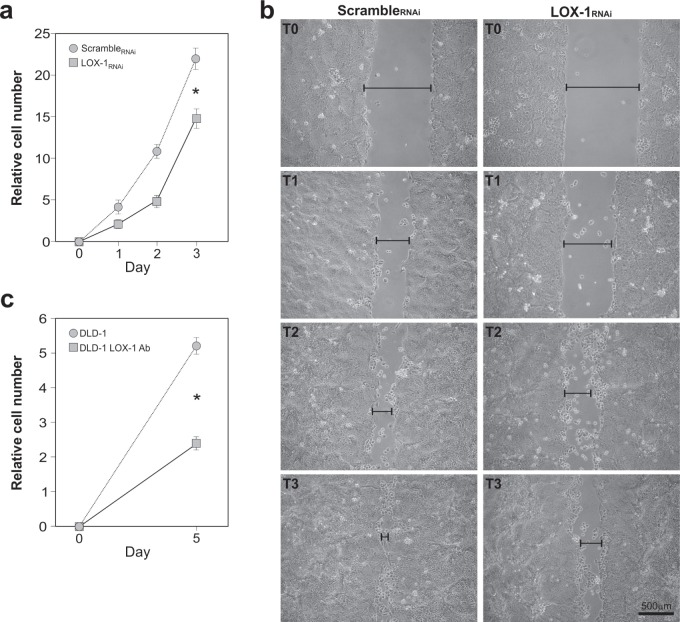
Effects of LOX-1 downregulation in DLD-1 cells **a**. Time course analyses of DLD-1 cell proliferation. Newly infected cells after puromycin selection were plated in replicates and daily counted. Growth curve of LOX-1_RNAi_ and scramble_RNAi_ DLD-1 cells was performed. The data represent the average ± S.D. of three separate experiments(*P <0.05). **b**. Wound-healing/invasion assay performed on LOX-1_RNAi_ and scramble_RNAi_ DLD-1 cells. After mechanically scratching cell monolayer, the timing of filling the gap was observed. Images were captured at 0 hour (T0), 24 hours(T1), 48 hours (T2) and 72 hours (T3) post wounding. The gap was indicated by a black bar. **c**. LOX-1 neutralization was performed by using a commercial antibody. Following 5 days, cells (DLD-1 LOX-1Ab) were collected, counted and compared to control ones (DLD-1). The assay reveals a strong reduction in DLD-1 cells proliferation (DLD-1 LOX-1Ab) compared to DLD-1 untreated cells (*P<0.05).

To confirm whether LOX-1 knockdown affects cell growth, we carried out time course analyses of DLD-1 cell proliferation. To do so, newly infected cells were first selected with puromycin, plated in replicates and daily counted. Figure 4a reports the growth curve of LOX-1_RNAi_ cells compared to scramble_RNAi_ DLD-1 cells. As shown, the depletion of LOX-1 expression results in cell proliferation slowdown, suggesting that LOX-1 is required in DLD-1 cell growth, as already reported in other cancer cell models [2].

Thus, we employed the wound-healing assay for analyzing additional potential changes of cancer cell phenotypes. In particular, we studied the motility of colon cancer cells following LOX-1 inhibition and estimated their migration rate (Figure 4b). DLD-1cells were infected and selected as previously reported, and then plated to obtain a homogeneous monolayer. According to standard procedures, scramble_RNAi_ and LOX-1_RNAi_ DLD-1 cells were mechanically displaced by scratching a line through the layer and the open gap inspected over time (1, 2, and 3 days after the scratch, named in the Figure as T1, T2, T3 respectively), as the cells move in and fill the damaged area. As shown in Figure 4b, LOX-1_RNAi_ DLD-1 cells display an impairment of the capacity in filling the damaged area (see T3 panel), when compared to scramble_RNAi_ DLD-1 cells.

In order to verify whether the over described changes due to the interfering events were cell line- dependent, we investigated some of the over mentioned experimental aspects in not metastatic HCT8 cells. As shown in Supplemental data Figure 1a, a strong reduction of LOX-1 transcript was evidenced by RT-qPCR in LOX-1_RNAi_ HCT8 cells. Also the growth curve analysis showed a reduction in terms of proliferation rate in LOX-1_RNAi_ HCT8 cells (Supplemental data Figure 1b), when compared to scramble_RNAi_. Wound-healing assay was also performed and, as shown in Supplemental data Figure 1c, an impairment in closing the scratch was observed in LOX-1_RNAi_ HCT8 cells, indicating that also HCT8 colorectal cell line resulted to be sensitive to LOX-1 depletion.

### Antibody-mediated LOX-1 neutralization inhibits cell growth in DLD-1 colon cancer cells

In order to verify whether interfering with LOX-1 function produces effects similar to those observed by the use of shRNAi, a polyclonal neutralizing anti-LOX-1 antibody was added to culture medium of an equal number of non-infected DLD-1 cells, as shown in Figure 4c. Following 5 days from antibody exposure, cells were collected, counted and compared to untreated cells. A marked decrease of the proliferation rate was observed after using anti-LOX-1 antibodies at 2.5 μg/ml (see DLD-1 LOX-1 Ab in Figure 4c).

### Volatile compounds analysis

The volatile compounds released from scramble_RNAi_ and LOX-1_RNAi_ DLD-1 cells were sampled once a day for the first three days of cell culture. A scrutiny of the chromatograms indicated that seven compounds were found in at least 3 samples (Table 2).

**Table 2:** List of the volatile compounds found in the headspace of Scramble _RNAi_ and LOX-1_RNAi_ DLD-1 cells

N°	Retention time [min]	Similarity [%]	Molecular weight [amu]	OCCURRENCE [%] (4 days)		Putative Name
**1**	13.34	94%	134	Scramble_RNAi_ DLD1	100%	2-Ethyl-1-hexanol
				LOX-1_RNAi_ DLD1	100%	
**2**	20.97	85%	158	Scramble_RNAi_ DLD1	0%	Butyrate compound
				LOX-1_RNAi_ DLD1	100%	
**3**	21.44	86%	128	Scramble_RNAi_ DLD1	33%	2,4,4-trimethyl-hexane
				LOX-1_RNAi_ DLD1	66%	
**4**	24.97	89%	142	Scramble_RNAi_ DLD1	100%	2,5,5-trimethyl-heptane
				LOX-1_RNAi_ DLD1	100%	
**5**	26.62	90%	184	Scramble_RNAi_ DLD1	66%	2,3,8-trimethyl-decane
				LOX-1_RNAi_ DLD1	66%	
**6**	28.16	91%	184	Scramble_RNAi_ DLD1	100%	3,8-dimethyl-undecane
				LOX-1_RNAi_ DLD1	100%	
**7**	29.17	81%	278	Scramble_RNAi_ DLD1	100%	Diheptyl-phthalate
				LOX-1_RNAi_ DLD1	100%	

The retention time is the time at which the compound related peak appears; the similarity is the score of identification made with library comparison; the molecular weight is reported since it provides a figure of the volatility of the compound; the occurrence column represents the presence (%) of each compound in the sample; the final column reports the putative name of the identified molecule.

For each compound in Table 2 the similarity score between measured and reference mass spectra is reported to be above 80%. 2-Ethyl-1-hexanol was found in the headspace of cultured lung cancer cells [29] and 3,8-dimethyl-undecane was identified in the breath of lung cancer affected individuals [30]. Methylated alkanes (2,4,4-trimethyl-hexane, 2,5,5-trimethyl-heptane,2,3,8-trimethyl-decane) are often found in cancer, and are typically products of oxidative stress [31].

**Figure 5:**
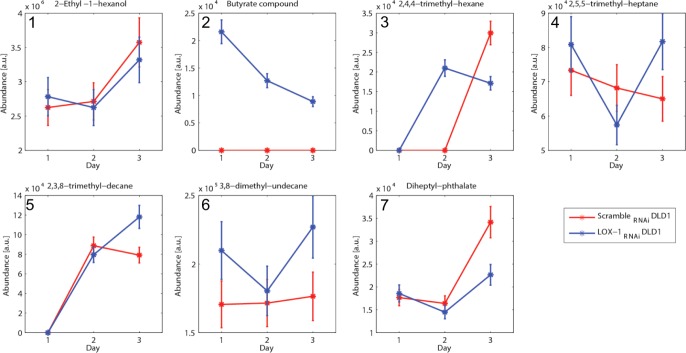
VOCs analysis and H4 acetylation pattern in DLD-1 cells **a.** Quantitative evaluation of the seven volatile compounds detected (listed in Table 2) vs. the days of growth. The compounds were sampled once a day for the first three days of cell culture. Seven of them were found in at least 3 samples, and in particular the compound number 2 (butyrate compound) is exclusively detected in LOX-1_RNAi_ DLD-1 cells. The abundance is calculated integrating the gas-chromatograms peaks.

The analysis of the amount of each compound observed at day 1, 2 and 3 of culture in scramble _RNAi_ and LOX-1_RNAi_ DLD-1 cells was reported in Figure 5. Almost all compounds showed an increase during days of culture, becoming more evident at the third day. Among these compounds it is important to note the behavior of the peak listed as butyrate compound that is exclusively found in the headspace of the LOX-1_RNAi_ DLD-1 cells (see Panel 2). This is the only compound whose abundance progressively decreases with the culture days. A previous analysis of the mass spectra identified this compound as butyric anhydride. Since this compound is a putative specific identifier of LOX-1_RNAi_ DLD-1, we investigated it in more detail, comparing the mass spectra with a reference standard. The results showed that the retention time of butyric anhydride peak is 14.85 min, but an additional peak was found at exactly 20.97 min. This result suggests that the compound is likely the product of reaction of the butyric anhydride with some molecules present in the culture medium, giving a butyrate derivative. The fragmentation of this compound in the mass spectrum leads to its identification as butyric anhydride by the instrument library.

This experiment clearly evidenced that scramble_RNAi_ and LOX-1_RNAi_ DLD-1 cell lines produced a distinct and recognizable pattern of VOCs, and specifically a butyrate compound is exclusively observed in LOX-1_RNAi_ DLD-1 cells.

**Figure 6:**
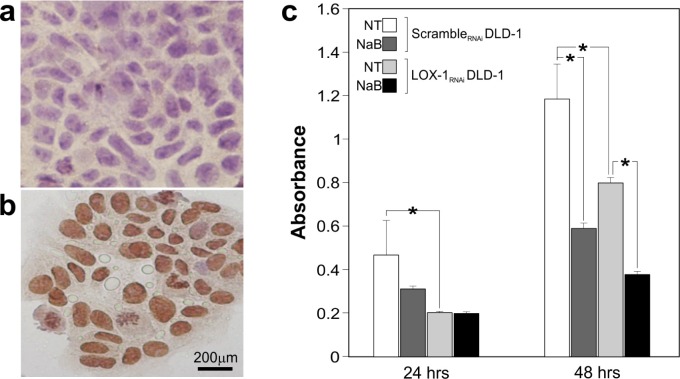
H4 acetylation immunocytochemistry and sodium Butyrate treatment **a.** H4 acetylation was evaluated by immunocytochemistry comparing scramble_RNAi_ and (**b**) LOX-1_RNAi_ DLD-1 cells. A strong increase of nuclear signal was observed in LOX-1_RNAi_ DLD-1 cells. Cells were light counterstained with eosin. 40X magnification. **c.** Na Butyrate treatment in scramble_RNAi_ and LOX-1_RNAi_ DLD-1 cells at 24 and 48 hours. The proliferation rate of scramble_RNAi_DLD-1 cells-treated with Na Butyrate- resulted to be slowed down both after 24 and 48 hours. Na Butyrate treatment to LOX-1_RNAi_ DLD-1 cells induced after 48 hours an evident decrease in term of proliferation rate respect to untreated ones.

Considering the involvement of butyrate in epigenetic regulation of tumor suppressor transcription via acetylation and its use as antineoplastic compound, we tested the level of histone H4 acetylation, representative of the acetylation pattern of genomic DNA, both in scramble_RNAi_ and LOX-1_RNAi_ DLD-1 by immunocytochemisty. As shown in Figure 6a, the Histone H4 acetylation is completely absent in scramble_RNAi_ cells, while is strongly evident in LOX-1_RNAi_ DLD-1cells (Figure 6b), further confirming the characterization made by VOC analysis.

Given the epigenetic effect of LOX-1 inhibition on histone H4 acetylation, we wondered if a well known hyperacetylating agent, such as Na butyrate, could play an interfering or synergizing role with LOX- 1 depletion in terms of proliferative rate. Therefore LOX-1_RNAi_ and scramble_RNAi_ DLD-1 cells were treated with Na Butyrate at a medium dose 5mM for 24 and 48 hours.

While no effect on proliferation was observed when Na Butyrate was added for 24 hours in LOX-1_RNAi_ DLD-1cells, after 48 hours the treatment was efficacious displaying about 50% of proliferation inhibition, when compared to untreated cells (LOX-1_RNAi_ DLD-1 NT; Figure 6c). These interesting results suggested that Na butyrate exerted a synergic effect with LOX-1 depletion on the proliferation rate. When performed in scramble_RNAi_ DLD-1 cells, the treatment seemed to display an impairment of the proliferation rate both after 24 and 48 hours, as compared to scramble untreated cells (scramble_RNAi_ DLD-1 NT).

## DISCUSSION

CRC is the third most common cause of cancer mortality in both men and women in United States [32]. Only a third of all CRC cases are diagnosed in the early stages and, despite advances in imaging, surgical techniques and neoadjuvant chemoradiotherapy, few patients with advanced CRC can be cured with synthetic therapy. Thus, it is necessary to discover the molecular mechanisms driving CRC progression for exploring more effective treatments.

Increased CRC risk is associated with obesity, diabetes, high cholesterol and atherosclerosis [2, 3], and individuals with high levels of circulating ox-LDL have a higher risk of CRC [26]. Ox-LDL receptor LOX-1 results to be involved in several mechanisms linked to tumorigenesis, in fact its altered expression has been associated besides to atherosclerosis, obesity, inflammation also to tumor development [2, 13, 33]. Once activated by ox-LDL, LOX-1 receptors stimulate the expression of adhesion molecules, proinflammatory signaling pathways and proangiogenic proteins [34], including VEGF. In addition, CRC tissue produces and accumulates an excess of ox-LDL, suggesting a close correlation between lipid dysfunction and malignant transformation [5, 6].

Preliminary meta-analysis data on LOX-1 mRNA expression profiles reported an upregulation of LOX-1 in 20% of colon cancer cell lines, suggesting its potential involvement in colon tumorigenesis [2]. Our observations obtained by immunohistochemistry on human tissues point out that LOX-1 protein expression is modulated in colon tumorigenesis, in fact we find a strong up-regulation of LOX-1expression in colon cancer tissues, as compared to healthy counterpart of the same patients. LOX-1 expression is modulated since the early stage of tumor development, suggesting a potential role in the insurgence and progression of the disease. Moreover, data obtained from human tissues indicate that LOX-1 expression correlates with the aggressiveness of the tumor, since it results significantly overexpressed in 72% G1-G2; any T; N0 and in 90% G2-G3; any T; N1,N2,M0,M1 colon cancer, when compared to normal tissue of the same patient.

To further demonstrate the role of LOX-1 in colon cancer, we designed a study to modulate its expression *in vitro* and to evaluate a possible influence of LOX-1 levels on the neoplastic phenotype of two CRC cell lines, DLD-1 and HCT8, showing a different grade of differentiation and aggressiveness.

The proliferation rate results to be significantly lowered in LOX-1_RNAi_ DLD-1 when compared to scramble_RNAi_ cells. Similarly, *in vitro* inhibition of LOX-1 receptors by using neutralizing antibodies demonstrates the involvement of LOX-1 in cell proliferation. Consistent with our findings, it was reported that up-regulation of LOX-1 by ox-LDL induce tumor angiogenesis and stimulate cell proliferation in prostate cancer cells, demonstrating a direct relationship between obesity factors and the enhancement of expression of proliferation and pro-angiogenic markers [35].

The wound-healing assay reveals an evident impairment of LOX-1 depleted cells in closing the scratch. These findings may be related to the role attributed to LOX-1 as adhesive molecule that can induce the transendothelial migration of the tumor cells, by interacting with phosphatidylserine expressed on their membrane [36].

Finally an innovative protocol was adopted for evaluating volatile compounds in scramble_RNAi_ and LOX-1_RNAi_ DLD-1cells. The analysis of volatile compounds (VOCs) is emerging as an attractive methodology to diagnose diseases and in particular cancer [37]. Some studies evidenced that VOCs in breath can differentiate between colon cancer and healthy subjects [29]. A number of studies showed the existence of specific patterns of volatile compounds that allows for the discrimination between cancer and normal cells [30]. Our results report a marked difference in VOC profile and, in particular, a butyrate compound seems to be secreted exclusively by LOX-1_RNAi_ DLD-1 cells. Butyrate has been shown to be protective against colorectal neoplasia. It can interfere with human colon cancer cells, by inhibiting cell proliferation and differentiation enhancing IkB-α degradation and inducing apoptosis targeting histone deacetylases [38–41]. Interestingly, downregulation of LOX-1 expression in DLD-1 cells strongly influences butyrate presence and consequently results in a marked increase of the histone H4 acetylation pattern. The shifting of acetylation pattern in LOX-1_RNAi_ DLD-1 compared to scramble_RNAi_ cells suggests a possible involvement of LOX-1 in epigenetic regulation of tumor suppressor genes. Although the molecular mechanism of action has to be fully elucidated, the hyper-acetylating condition due to the addition of sodium butyrate seems to cooperate in the amplification of LOX-1 silencing anti-proliferative action, suggesting a synergetic action. While the effect of sodium Butyrate on scramble_RNAi_ DLD-1 cell proliferation is evident already after 24 hours, in LOX-1 deficient cells (LOX-1_RNAi_ DLD-1) it become appreciable only after 48 hours, probably due to their slower proliferation rate.

Thus, VOC analysis *in vivo* could be considered a noninvasive method for indicating the presence of compounds possibly modulated by LOX-1 expression and related to colon tumorigenesis. Such a technique may also allow non-invasive monitoring of response to therapy and could revolutionize screening practices for colorectal cancer and potentially many other gastrointestinal diseases.

Altogether, these results suggest that LOX-1 may act as a molecular link among metabolism, inflammation and cancer, indicating its potential role as biomarker and new molecular target, and representing an attractive and concrete opportunity to improve current therapeutic strategies for CRC.

## MATERIALS AND METHODS

### Patient characteristics and tissue samples

Twenty-eight colorectal adeno-carcinomas (CRC) were collected irrespectively of the clinical staging. The mean age of the patients at the time of surgery was 69±5 and the male/female ratio was 2.6. All specimens used in this study underwent histological examination to confirm the diagnosis. None of the patients had received any therapy before surgery. Moreover, normal mucosa from healthy people (n=8) was collected from autoptic examination (performed within six hours from death), resulting negative for any neoplasia and gastrointestinal diseases at the macroscopic evaluation of whole gastrointestinal apparatus and was used as control. Distant normal mucosa (NM, n=28) and adenomas with low grade dysplasia (ALGD, n=8) were collected and analyzed. Histological classification was carried out on haematoxilin and eosin-stained slides.

### Immunohistochemistry (IHC) and Immunocytochemistry (ICC)

Serial four micron sections from formalin-fixed and paraffin embedded specimens were immunostained for LOX-1 following the streptavidin-biotin method, as previously described [27]. In brief, sections were deparaffinized and rehydrated in decreasing alcohol concentration. Tissue antigen retrieval was performed in citrate buffer by microwave. Endogenous peroxidase activity was quenched with 0.03% hydrogen peroxide in absolute methanol, for 30 min at room temperature. The primary antibody used was rabbit polyclonal anti-LOX-1 (H19 Santa Cruz Biotechnology Inc. CA 95060, USA) validated for immunohistochemical analysis (1:50). Biotinylated anti-goat IgG (Dako A/S, Denmark) was used as secondary antibody. After washing, sections were treated with streptavidin-peroxidase reagent (Dakopatts A\S, Denmark), incubated with diaminobenzidine (DAB) and lightly counterstained with hematoxylin. Slides were independently examined by two pathologists, unaware of the clinical data and molecular results. Tissue staining was semi-quantitatively graded for intensity as negative/weak: 0, moderate: 1 and strong: 2. The cell positivity was also scored as less than 10% (0), from 10% to 25% (1) from 26% to more than 50% (2). The final score was calculated by adding both partial scores. For evaluation of results, Chi square test was performed. For immunocytochemistry, DLD-1 colon cancer cells were plated in 4 wells/chamber slides at a concentration of 30.000 x cm^2^. At the end, growth medium was removed and cells were rinsed in phosphate-buffered saline (PBS) and fixed in formalin 10% solution (Sigma-Aldrich, St.Louis, MO, USA) for 5 min. Then cells were permeabilized with 0,5% Triton X-100 and 0.05% Tween-20 in PBS. For LOX-1 detection, the anti-LOX-1 primary antibody (H19 Santa Cruz Biotechnology Inc. CA 95060, USA) was used at the dilution 1:80 and the rabbit polyclonal Biotinylated anti-goat IgG (Dako A/S, Denmark) as secondary antibody. Detection of Acetylated histone H4 was performed using rabbit polyclonal antibody 1:400 (Upstate Biotecnology, Lake Placid, NY 12946)

### Cell cultures

Human colon cancer cell lines: DLD-1 (ATCC: CCL-221^TM^), LoVo (ATCC: CCL-229^TM^), HCT8 (ATCC: CCL-244^TM^), SW480 (ATCC: CCL-228^TM^) were used. mRNA expression analyses were performed on DLD-1, HCT8, LoVo and SW480 cell lines. HCT8 cells were used in RNAi experiments, proliferation rate and wound healing assay. DLD-1 were employed in all the experiments mentioned in the manuscript. Human colon cancer cell lines and kidney HEK-293T cells were grown in RPMI-1640 (Gibco, Life Technologies Corporation, Carlsbad, CA, USA) and Dulbecco's Modified Eagle Medium (DMEM, GE healthcare, Milan, IT), respectively. RPMI-1640 was supplemented with 15% fetal bovine serum (FBS) (Euroclone, Milan, IT), Glutamine (Euroclone, Milan, IT), non-essential Amino Acids (Gibco, Life Technologies Corporation, Carlsbad, CA, USA), Penicillin-Streptomycin (Gibco, Life Technologies Corporation, Carlsbad, CA, USA). DMEM medium was completed with 10% FBS, Glutamine, Penicillin-Streptomicin, (Euroclone, Milan, IT). Human colon cancer cell lines were kindly gifted by G. Casey (USC Norris Comprehensive Cancer Center, Los Angeles, USA).

### Lentiviral vectors

To develop lentiviral vectors suitable to knockdown LOX-1 mRNA, synthetic oligonucleotides (Integrated DNA Technologies, Inc, Coralville, Iowa, US) were designed and opportune restriction site included. We developed a strategy to obtain bi-directional lentiviral vectors expressing short hairpin RNAs (shRNAs) against LOX-1 mRNA (*ShRNAi #3*: 5′-GGAAATGATAGAAACCCTTGC-3′, *ShRNAi #4*: 5′-GCACAGCTGATCTGGACTTCA-3′, *ShRNAi #5*:5′-GCCAAGAGAAGTGCTTGTCTT-3′) plus a scramble shRNA not targeting any human gene (*ShRNAi scramble*: 5′-GAACAAGATGAAGAGCACCTT-3′), under the control of H1 promoter, and the gene encoding puromycin resistance under the control of hPGK promoter. To do so, we first cloned into the pSuperior plasmid (Addgene Inc, Cambridge, MA, USA) synthetic oligonucleotides able to efficiently interfere with *orl1* transcript [2]. We then moved from pSuperior into the pRRL-SIN-cPPT-PGK/GFP-WPRE lentiviral backbone, a cassette containing the H1promoter, the shRNA, the hPGK promoter and the puromycin resistance gene (Addgene Inc, Cambridge, MA, USA). To avoid rearrangements, lentivectors were transformed into Stbl3 strain (Invitrogen, Life Technologies Corporation, Carlsbad, CA, USA).

### Lentiviral vectors productions

Production of lentiviral particles was performed cotransfecting HEK-293T cells with lenti-transfer vectors and the third generation of vector packaging mix (pLP-1, pLP2 and pLP-VSV-G, Invitrogen, Life Technologies Corporation, Carlsbad, CA, USA). Transfections were carried out using CalPhos transfection kit (Clontech Laboratories, Inc, Mountain View, CA). Forty-eight hours post-transfection, the culture media were collected, centrifuged at 3000rpm for 10 minutes and the supernatants filtered. For cell infection, supernatant were incubated for 12 hours, then the culture medium was removed and fresh RPMI medium supplemented with 15%FBS was added to cells. Puromycin was used (5 mg/ml; Sigma-Aldrich, St Louis MO, USA) to counterselect infected cells.

### Gene expression analyses

For stabilizing LOX-1 expression, a medium refresh was made 16 h before assessing mRNA levels in order to give back consistent RT-qPCR measures. Total RNAs from cells and tissues were extracted by Trizol Reagent (Invitrogen Life Technologies Corporation, Carlsbad, CA, USA) following manufacturer's instructions. Treatment with DNase I-RNase-free (Ambion, Life Technologies Corporation, Foster City, CA, USA) was used to eliminate genomic DNA contamination from total RNA samples. One μg of RNA was reverse transcribed with the High-Capacity cDNA Archive kit (Life Technologies Corporation, Foster City, CA, USA) and used in RT-PCR and RT-qPCR. mRNAs were measured by SYBR Green (Life Technologies Corporation, Foster City, CA, USA) using the following primers:

LOX-1: forward 5′-AAACCCTTGCTCGGAAGCTGAA-3′,

LOX-1: reverse 5′-TGCGGACAAGGAGCTGAACAAT-3′,

GAPDH: forward 5′-TTGCCCTCAACGACCACTTTG-3′,

GAPDH: reverse 5′-CACCCTGTTGCTGTAGCCAAATTC-3′.

GAPDH was used as reference gene. The comparative ΔΔCt method was used to quantify relative gene expression levels.

### Purification of caveolae-enriched membrane fractions and Western blot analysis

Caveolae-enriched membrane fractions were prepared by a detergent-free purification, as previously described [21]. Two confluent 90-mm dishes of DLD-1were lysed in 500 mM sodium carbonate, pH 11, containing proteases inhibitor cocktail set III (0.1 mM AEBSF hydrochloride, 0.5 uM aprotinin, 5 mM Bestatin, 1.5 uM E-64, 10 uM Leupeptin, 1 mM Pepstatin A) (Calbiochem, La Jolla, CA, USA) and 1 uM phenylmethylsulfonyl fluoride (PMSF) (Euroclone, Devon, UK), homogenized and sonicated. A 5-45% discontinuous sucrose density gradient was formed in 25 mM Mes, pH 6.5, 0.15 M NaCl and centrifuged at 39,000 rpm for 16-20 h in an SW41 rotor (Beckman Instruments, Palo Alto, CA). Samples were fractionated in 1 ml aliquots from the top to the bottom. Protein concentration was measured in each fraction by Bradford assay (Sigma-Aldrich, St. Louis, MO). Proteins from each fraction were precipitated with 10% trichloroacetic acid (TCA), solubilized in SDS-PAGE sample buffer. Fractions were analyzed in 10% acrylamide gels and transferred to polyvinylidenedifluoride (PVDF) membranes (GE Healthcare, Chalfont St. Giles, UK) for 30 minutes at 15 V (Semi-Dry Transfer cell, Biorad, Hercules, CA). Western blot was carried out using anti-LOX-1 (Ox-LDL R-1 Antibody E-19 -Santa Cruz Biotechnology, CA USA) and anti-flotilin antibodies(Santa Cruz Biotechnology). Immunoreactive bands were visualized by enhanced chemiluminescence (ECL, Sigma-Aldrich, St. Louis, MO).

### Cell proliferation assays

Cells were seeded at the density of 25,000 cells/cm^2^. Individual cell lines were collected and cell number determined by hemocytometric counting for three consecutive days. Cell numbers were normalized to day 0 values. The count has been repeated three times and evaluated by two different operators. Treatment with Na Butyrate: LOX-1_RNAi_ DLD-1 and scramble_RNAi_ DLD-1 were seeded at the density of 5,000 cells/cm^2^ in a 96-well plate. After 24 hours Na Butyrate (Sigma-Aldrich S.r.l., Milan, Italy) was added at the concentration of 5mM for 24 and 48 hours. Cells cultured in the absence of Na Butyrate was used as control, and MTT assay was performed to determine cell viability. After removing the supernatant of each well and washing twice by PBS, 20 μl of MTT solution (5 mg/ml in PBS) and 100 μl of medium were added. After three hours, the resultant formazan crystals were dissolved in dimethyl sulfoxide (100 μl) and the absorbance intensity was measured by a microplate reader (Bio-RAD 680, USA) at 490 nm with a reference wavelength of 620 nm. All experiments were performed in triplicate.

### Cell motility analysis

Wound healing assay was performed to evaluate the cell motility. Cells were grown in six-well dishes at 1×10^6^ cells/well. A single scratch wound was created using a p10 micropipette tip into confluent cells. Cells were washed with PBS for three times to remove cell debris, assay medium was added. Microimages of the scratches were taken under a microscope at 0h (control), and 24h, 48h and 72h after the scratch, and the distance of the gaps was evaluated at different time points [28]. Images were captured by Nikon Eclipse TE2000S inverted optical microscope (10X magnification).

### Antibody-mediated neutralization

DLD-1 cells were incubated with or without anti-LOX-1 antibody (Ox-LDL R-1 Antibody E-19 -Santa Cruz Biotechnology, CA USA) added to culture medium at a concentration of 2.5 μg/ml. Following 5 days exposure, cells were collected, counted and compared to control cells. No detectable change was observed with lower antibody concentrations (data not shown).

### Volatile compounds analysis

The volatile compounds released by cell cultures were sampled and then analyzed with a gas-chromatography mass-spectrometer (GC-MS) equipment (GCMS-QP 2010 Shimadzu). To collect the volatile compounds special lids in polymethylmethacrylate (PMMA) to fit with Petri dishes were designed. The lids were endowed with a fixture suitable to be connected with the holder of a solid phase micro-extraction (SPME) fiber. SPME is a standard sampling method in VOCs analysis that allows for an efficient collection and storage of volatile compounds. The fibers used in this experiment were coated with 50/30 μm Divinylbenzene/Carboxen/PDMS (SUPELCO, Bellefonte, PA, USA). The fibers were exposed to the cells headspace for 1 h. During the exposure the cultures were kept in the incubator at a temperature of 37°C and at a CO_2_ concentration of 5%.

The samples collected in the SPMEs were analyzed the same day of collection with a GCMS-QP 2010 Shimadzu series gas chromatograph mass spectrometer, equipped with an EQUITY-5 capillary column (poly(5% diphenyl/95% dimethyl siloxane) phase (SUPELCO, Bellefonte, PA, USA). The column was 30 m length × 0.25 mm I.D.× 0.25 μm thickness.

The volatile compounds adsorbed in the SPME were desorbed from the fiber at 250°C for 3 minutes in the GC injection port. The volatile organic compounds were separated on the GC column using an initial oven temperature of 40°C for 5 minutes, then increased by 7°C/min to 220°C, a second temperature increase by 15°C/min to 300°C that was held for 3min (total runtime: 39 min). The analyses were performed in splitless mode, using ultra-high purity helium as carrier gas.

The instrument was operated in linear velocity constant mode, with a carrier gas pressure of 24.9 kPa, total flow of 5.9 mL/min, column flow of 0.7 mL/min and linear velocity of 30.2 cm/s.

The mass spectrometer was a single quadrupole analyzer in electronic ionization mode, scanned over a mass range of m/z 40-450 amu in the full scan mode. The detector voltage was fixed at 0.7 kV. The temperature of interface and ion source was kept constant at 250°C. The GC-MS data were analyzed using the section GCMS post-run analysis of the GCMS solutions software (version 2.4, Shimadzu Corporation). Tentative identification of compounds was done using both NIST 127 and NIST 147 libraries. For the identification of a specific compound butyric anhydride (purum, ≥97% (NE), Fluka Analytical, Italy) was used as reference standard, spiking the DLD1 cell culture medium.

For investigating butyric anhydride in more detail, it has been dissolved in culture medium at a final concentration of 0.1%. The solution, kept at 37°C in a thermal bath was analyzed by VOCs in the headspace sampled by SPME with an exposure time of 1h.

### Statistical analysis

All values provided in the text and figures are means of three independent experiments ± standard deviations (SD). Mean values were compared using the two-tailed Student t-test, for independent samples. For LOX-1 immunohistochemistry Chi square test was used to compare normal mucosa with adenomas and adenocarcinomas. For volatile compounds analysis descriptive statistical analysis have been performed with Matlab (The Math Works, Inc., Natick, Ma, USA).

## SUPPLEMENTARY FIGURE



## Abbreviations

ALGD: adenoma with low grade dysplasia
CRC: colorectal cancer
CTLD: lectin-like extracellular C-terminal domain
DAB: diaminobenzidine
DMEM: Dulbecco's Modified Eagle Medium
FBS: fetal bovine serum
GAPDH: Glyceraldehyde 3-phosphate dehydrogenase
GC-MS: chromatography-mass spectrometry
GCMS: gas-chromatography mass-spectrometer
GCMS-QP: gas-chromatography mass-spectrometer equipment
HEK: human embryonic kidney
ICC: Immunocytochemistry
IHC: Immunohistochemistry
iKB-α: nuclear factor of kappa light polypeptide gene enhancer in B-cells inhibitor, alpha
LOX-1: lectin-like oxidized LDL receptor-1
mRNA: messenger RNA
MTT: tetrazolium dye MTT 3-(4,5-dimethylthiazol-2-yl)-2,5-diphenyltetrazolium bromide
Na B: sodium Butyrate
NF-kB: nuclear factor kappa-light-chain-enhancer of activated B cells
NIST: National Institute of Standards and technology
NM: normal mucosa
NO: nitric oxide
ox-LDL: oxidized low density lipoprotein
PBS: phosphate-buffered saline
PDMS: polydimethylsiloxane
PMMA: polymethylmethacrylate
PMSF: phenylmethylsulfonyl fluoride
PVDF: polyvinylidenedifluoride
RNAi: RNA interference
ROS: reactive oxygen species
RPMI: Roswell Park Memorial Istitute
RT-PCR: real time-polymerase chain reaction
RT-qPCR: real time-quantitative polymerase chain reaction
SD: standard deviation
SDS-PAGE: Sodium Dodecyl Sulphate - PolyAcrylamide Gel Electrophoresis
shRNAs: short hairpin RNAs
SMC: smooth muscle cell
SNPs: single nucleotide polymorphisms
SPME: solid phase micro-extraction
TCA: trichloroacetic acid
VEGF: vascular endothelial growth factor
VOCs: volatile organic compounds
WHO: world health organization

## References

[1] Li CJ, Zhang X, Fan GW (2014). Updates in colorectal cancer stem cell research. J Cancer Res Ther.

[2] Hirsch HA, Iliopoulos D, Joshi A, Zhang Y, Jaeger SA, Bulyk M, Tsichlis PN, Shirley Liu X, Struhl KA (2010). Transcriptional signature and common gene networks link cancer with lipid metabolism and diverse human diseases. Cancer Cell.

[3] Morganti M, Carpi A, Nicolini A, Gorini I, Glaviano B, Fini M, Giavaresi G, Mittermayer Ch, Giardino R (2002). Atherosclerosis and cancer: common pathways on the vascular endothelium. Biomed Pharmacother.

[4] Qi XF, Kim DH, Yoon YS, Kim SK, Cai DQ, Teng YC, Shim KY, Lee KJ (2010). Involvement of oxidative stress in simvastatin induced apoptosis of murine CT26 colon carcinoma cells. Toxicol Lett.

[5] Keshavarzian A, Zapeda D, List T, Mobarhan S (1992). High levels of reactive oxygen metabolites in colon cancer tissue: analysis by chemiluminescence probe. Nutr Cancer.

[6] Otamiri T, Sjodahl R Increased lipid peroxidation in malignant tissues of patients with colorectal cancer. Cancer.

[7] Lu J, Mitra S, Wang X, Khaidakov M, Mehta JL (2011). Oxidative stress and lectin like ox-LDL receptor LOX-1 in atherogenesis and tumorigenesis. Antioxid Redox Signal.

[8] Poynter JN, Gruber SB, Higgins PD, Almog R, Bonner JD, Rennert HS, Low M, Greenson JK, Rennert G (2005). Statins and the risk of colorectal cancer. N Engl J.

[9] Chan AO, Jim MH, Lam KF, Morris JS, Siu DC, Tong T, Ng FH, Wong SY, Hui WM, Chan CK, Lai KC, Cheung TK, Chan P (2007). Prevalence of colorectal neoplasm among patients with newly diagnosed coronary artery disease. JAMA.

[10] Aoyama T, Sawamura T, Furutani Y, Matsuoka R, Yoshida MC, Fujiwara H, Masaki T (1999). Structure and chromosomal assignment of the human lectin-like low density-lipoprotein receptor-1(LOX-1) gene. Biochem J.

[11] Mango R, Biocca S, delVecchio F, Clementi F, Sangiuolo F, Amati F, Filareto A, Grelli S, Spitalieri P, Filesi I, Favalli C, Lauro R, Mehta JL (2005). *In vivo* and *in vitro* studies support that a new splicing isoform of OLR1 gene is protective against acute myocardial infarction. Circ Res.

[12] Biocca S, Filesi I, Mango R, Maggiore L, Baldini F, Vecchione L, Viola A, Citro G, Federici G, Romeo F, Novelli G (2008). The splice variant LOXIN inhibits LOX-1 receptor function through hetero-oligomerization. J Mol Cell Cardiol.

[13] Draude G, Hrboticky N, Lorenz RL (1999). The expression of the lectin-like oxidized low-density lipoprotein receptor (LOX-1) on human vascular smooth muscle cells and monocytes and its down-regulation by lovastatin. BiochemPharmacol.

[14] Mehta JL, Li D (2002). Identification regulation and function of a novel lectin-like oxidized low-density lipoprotein receptor. J Am CollCardiol.

[15] Chen M, Masaki T, Sawamura T (2010). LOX-1, the receptor for oxidized low-density lipoprotein identified from endothelial cells: implications in endothelial dysfunction and atherosclerosis. PharmacolTher2002generation, and infiammation in human endothelial cells.

[16] Ohki I, Ishigaki T, Oyama T, Matsunaga S, Xie Q, Ohnishi-Kameyama M, Murata T, Tsuchiya D, Machida S, Morikawa K, Tate S (2005). Crystal structure of human lectin-like, oxidized low-density lipoprotein receptor 1 ligand binding domain and its ligand recognition mode to OxLDL. Structure.

[17] Park H, Adsit FG, Boyington JC (2005). The 1. 4 angstrom crystal structure of the human oxidized low density lipoprotein receptor lox-1. J BiolChem.

[18] Falconi M, Biocca S, Novelli G, Desideri A (2007). Molecular dynamics simulation of human LOX-1 provides an explanation for the lack of OxLDL binding to the Trp150Ala mutant. BMC Struct Biol.

[19] Cao W, Calabro V, Root A, Yan G, Lam K, Olland S, Sanford J, Robak A, Zollner R, Lu Z, Ait-Zahra M, Agostinelli R, Tchistiakova L (2009). Oligomerization is required for the activity of recombinant soluble LOX-1. FEBS J.

[20] Ohki I, Amida H, Yamada R, Sugihara M, Ishigaki T, Tate S (2011). Surface plasmon resonance study on functional significance of clustered organization of lectin-like oxidized LDL receptor (LOX-1). BiochimBiophys Acta.

[21] Matarazzo S, Quitadamo MC, Mango R, Ciccone S, Novelli G, Biocca S (2012). Cholesterol-lowering drugs inhibit lectin-like oxidized low-density lipoprotein-1 receptor function by membrane raft disruption. MolPharm.

[22] Biocca S, Iacovelli F, Matarazzo S, Vindigni G, Oteri F, Desideri A, Falconi M (2015). Molecular mechanism of statin-mediated LOX-1 inhibition. Cell Cycle.

[23] Lee WJ, Ou HC, Hsu WC (1999). Ellagic acid inhibits oxidized LDL-mediated Lox-1 expression ROS receptor (LOX-1) on human vascular smooth muscle cells and monocytes and its down-regulation by lovastatin. BiochemPharmacol.

[24] Cominacini L, Pasini AF, Garbin U, Davoli A, Tosetti ML, Campagnola M, Rigoni A, Pastorino AM, Lo Cascio V, Sawamura T (2000). Oxidized low density lipoprotein (ox-LDL) binding to ox-LDL receptor-1 in endothelial cells induces the activation of NF-kappaB through an increased production of intracellular reactive oxygen species. J BiolChem.

[25] Cominacini L, Rigoni A, Pasini AF, Garbin U, Davoli A, Campagnola M, Pastorino AM, Lo Cascio V, Sawamura T (2001). The binding of oxidized low density lipoprotein (ox-LDL) to ox-LDL receptor-1 reduces the intracellular concentration of nitric oxide in endothelial cells through an increased production of superoxide. J Biol Chem.

[26] Suzuki K, Ito Y, Wakai K, Kawado M, Hashimoto S, Toyoshima H, Kojima M, Tokudome S, Hayakawa N, Watanabe Y, Tamakoshi K, Suzuki S, Ozasa K (2004). Japan Collaborative Cohort Study Group Serum Oxidized Low-Density Lipoprotein Levels and Risk of Colorectal Cancer: A Case-Control Study Nestedin the Japan Collaborative Cohort Study. Cancer Epidemiol Biomarkers Prev.

[27] Hsu SM, Raine L, Fanger H (1981). The use of antiavidinantiboy and avidin-biotin-peroxidase complex in immunoperoxidase technics. Am j ClinPathol.

[28] Hirsch HA, Iliopoulos D, Tsichlis PN, Struhl K (2009). Metformin selectively targets cancer stem cells, and acts together with chemotherapy to block tumor growth and prolong remission. Cancer Res.

[29] Barash O, Peled N, Hirsch F, Haick H (2009). Sniffing the unique odor print of non small cell lung cancer with gold nanoparticles. Small.

[30] Peng G, Tisch U, Adams O, Hakim M, Shehada N, Broza YY, Billan S, Abdah-Bortnyak R, Kuten A, Haick H (2009). Diagnosing lung cancer in exhaled breath using gold nanoparticles. Nature Nanotechnology.

[31] Hakim M, Broza YY, Barash O, Peled N, Phillips M, Amann A, Haick H (2012). Volatile organic compounds of lung cancer and possible biochemical pathways. Chem Rev.

[32] Siegel RL, Miller KD, Jemal A (2015). Cancer Statistics, 2015. CA Cancer J Clin.

[33] Khaidakov M, Mitra S, Kang BY, Wang X, Kadlubar S, Novelli G, Raj V, Winters M, Carter WC, Mehta JL (2011). Oxidized LDL Receptor 1(ORL1) as a possible link between Obesity Dyslipidemia and Cancer. PlosOne.

[34] Dandapat A, Iau C, Sun L, Mehta JL (2007). Small concentrations of ox-LDL induce capillary tube formation from endotelial cells via LOX-1 dependent redox-sensitive pathway. ArteriosclerThrombVasc Biol.

[35] González-Chavarría I, Cerro RP, Parra NP, Sandoval FA, Zuñiga FA, Omazábal VA, Lamperti LI, Jiménez SP, Fernandez EA, Gutiérrez NA, Rodriguez FS, Onate SA, Sánchez O (2014). Lectin-like oxidized LDL receptor-1 is an enhancer of tumor angiogenesis in human prostate cancer cells. PLoS One.

[36] Honjo M, Nakamura K, Yamashiro K, Kiryu J, Tanihara H, McEvoy LM, Honda Y, Butcher EC, Masaki T, Sawamura T (2003). Lectin-likeoxidized LDL receptor-1 is a cell-adhesion molecule involved in endotoxin-induced inflammation. Proc Natl Acad Sci USA.

[37] Krilaviciute A, Heiss JA, Leja M, Kupcinskas J, Haick H, Brenner H (2015). Detection of cancer through exhaled breath: systematic review. Oncotarget.

[38] Hague A, Manning AM, Hanlon KA, Huschtscha LI, Hart D, Paraskeva C (1993). Sodium butyrate induces apoptosis in human colonic tumor cell lines in a p53-independent pathway: implications for the possible role of dietary fiber in the prevention of large-bowel cancer. C. Int. J. Cancer.

[39] Heerdt BG, Houston MA (1994). & Augenlich LH. Potentiation by specific short-chain fatty acids of differentiation and apoptosis in human colonic carcinoma cell lines. Cancer Res.

[40] Vanhoutvin SA, Troost FJ, Hamer HM, Lindsey PJ, Koek GH, Jonkers DM, Kodde A, Venema K, Brummer RJ (2009). Butyrate-Induced Transcriptional Changes in Human Colonic Mucosa. PLoS ONE.

[41] Yoshida T, Sekine T, Aisaki K, Mikami T, Kanno J, Okayasu I (2011). CITED2 is activated in ulcerative colitis and induces p53-dependent apoptosis in response to butyric acid. Am J. of Gastroenterol.

