# Supplementary material for: The lectin-like oxidized LDL receptor-1: a new potential molecular target in colorectal cancer
Source: Oncotarget. 2016 Feb 17;7(12):14765–80. doi: 10.18632/oncotarget.7430 (PMC4924750; doi:10.18632/oncotarget.7430)
Supplement: Supplementary file 1 [file oncotarget-07-14765-s001.pdf]

SUPPLEMENTARY FIGURE

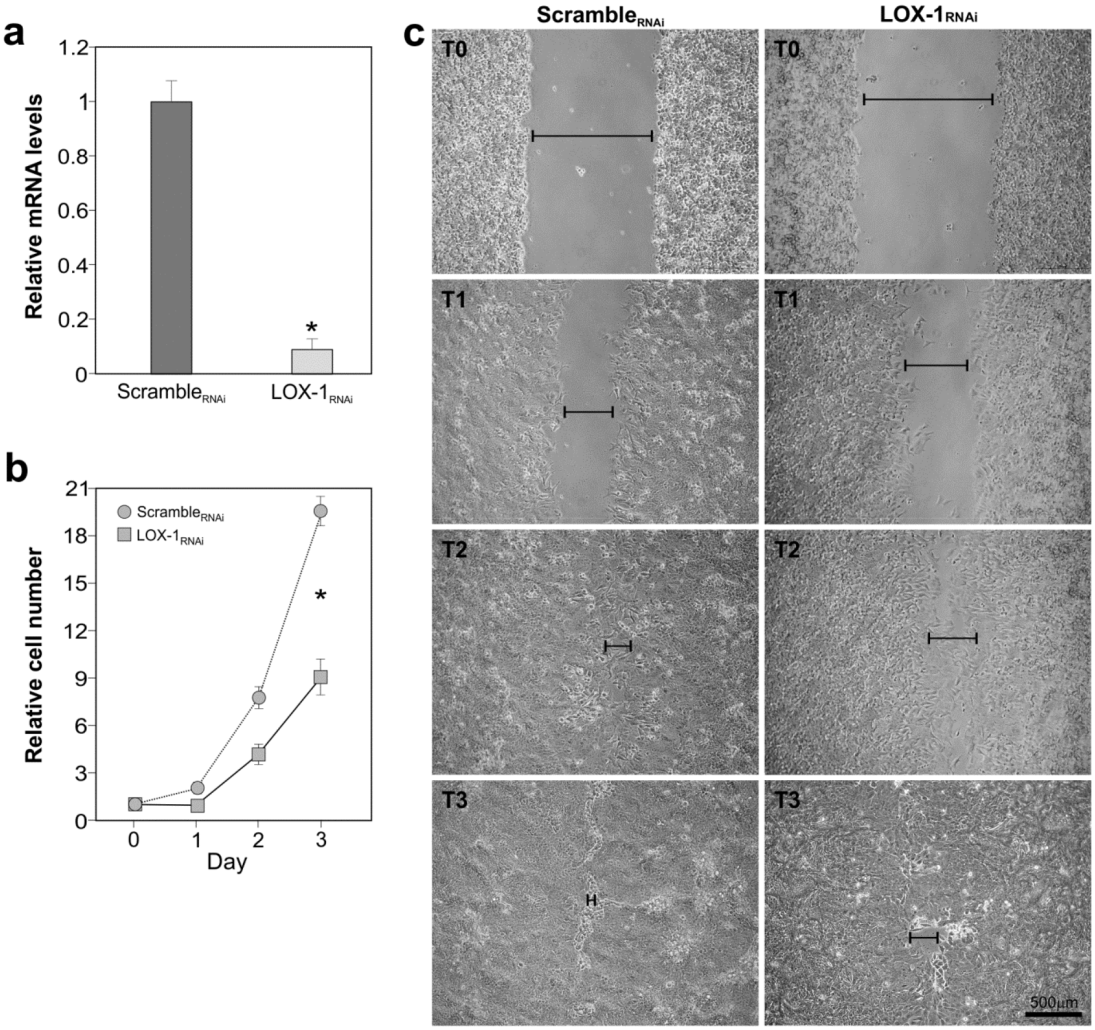

Supplementary Figure S1: Effects of LOX-1 downregulation in HCT-8 cells. a. RT-qPCR analyses of LOX-1 mRNA levels in LOX-1_{RNAi} and $Scramble_{RNAi}$ HCT-8 cells, 6 days post virus administration. Data are representative of three independent experiments and reported as mean ± SEM (*P<0,05). **b.** Growth curve of LOX-1_{RNAi} and $Scramble_{RNAi}$ HCT-8 cells; (*P<0,05). **c.** Wound healing/invasion assay performed on LOX-1_{RNAi} and $Scramble_{RNAi}$ HCT-8 cells. Images were captured at 0 hour (T0), and 24 hours(T1), 48 hours (T2) and 72 hours (T3) post wounding.
